# Homologous recombination deficiency and glycolysis‐related pathway in adjuvant chemotherapy for triple‐negative breast cancer: A genomic landscape and biomarker assessment of the PATTERN trial

**DOI:** 10.1002/ctm2.513

**Published:** 2021-09-26

**Authors:** Si‐Yuan Zhu, Ding Ma, Fu‐Gui Ye, Zhi‐Ming Shao, Ke‐Da Yu

**Affiliations:** ^1^ Department of Breast Surgery Fudan University Shanghai Cancer Center Shanghai China; ^2^ Shanghai Medical College Fudan University Shanghai China; ^3^ Shanghai Key Laboratory of Breast Cancer Shanghai China

**Abbreviations**
BCS: breast conservative surgery
BLIS: basal‐like and immune‐suppressed
CEF‐T: fluorouracil, epirubicin, and cyclophosphamide followed by docetaxel
FUSCC: Fudan University Shanghai Cancer Center
HRD: homologous recombination deficiency
IM: immunomodulatory
IQR: interquartile range
LAR: luminal androgen receptor
MES: mesenchymal‐like
PCb: paclitaxel and carboplatin

Dear Editor,

Triple‐negative breast cancer (TNBC) is associated with genome‐wide instability caused by mutations in homologous recombination repair mechanism,^1^ and the application of DNA‐damaging compounds has been explored for TNBC.^2^ Recently, we performed the PATTERN trial (NCT01216111) to compare six cycles of paclitaxel plus carboplatin (PCb) with a standard‐dose regimen of three cycles of cyclophosphamide/epirubicin/fluorouracil followed by three cycles of docetaxel (CEF‐T) in the adjuvant setting of early‐stage TNBC, and the result indicated a superior efficacy of the carboplatin‐containing regimen and good tolerance to both treatments.^3^ In this study, we conducted multi‐omic profiling on 132 patients in the PATTERN cohort to investigate potential biomarkers for a more precise choice of adjuvant chemotherapy regimen for TNBC. We found that homologous recombination deficiency (HRD) score might serve as a biomarker of adjuvant carboplatin‐containing regimen for TNBC, and upregulation of glycolysis and hypoxia‐related pathways might participate in underlying mechanisms of anthracycline/taxane‐based regimen resistance.

The abovementioned 132 patients in the PATTERN cohort have been enrolled into the Fudan University Shanghai Cancer Center Triple‐Negative Breast Cancer (FUSCC‐TNBC) program to receive whole‐exome sequencing, RNA sequencing, and copy number detection.^4^ We investigated the association of multi‐omic data with relapse‐free survival (RFS) to explore potential biomarkers. Figure S1 shows the distribution of cases enrolled. Table S1 demonstrated characteristics of the PATTERN cohort and the patients involved. Additional transcriptomic data of 165 TNBC patients who received anthracycline/taxane‐based chemotherapy in the Molecular Taxonomy of Breast Cancer International Consortium (METABRIC) database were analyzed for external validation. Table S2 illustrates the characteristics of these patients.

Clinicopathologic and molecular characteristics were similar between the two arms (Figure 1 and Table 1). FUSCC subtype composition of this cohort was similar to that of the whole FUSCC‐TNBC cohort (Figure 1A). TP53 (76.4%), PIK3CA (18.0%), KMT2C (9.0%), PTEN (6.7%), and NF1 (5.6%) were the most frequently mutated genes (Figure 1B). HRD‐related signatures (signature 3 and 8) and clock‐like signatures (signature 1 and 5) were the dominant mutational signatures (Figure 1C).^5^, ^6^ Furthermore, 115 cases in this cohort with copy number‐based HRD score, which has been reported to be a potential predictor for a platinum‐containing regimen, had a median value of 26.0 (Figure 1D).^7^

**FIGURE 1:**
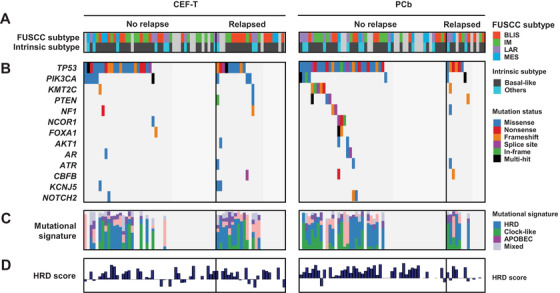
Genomic landscape by treatment cohorts. (A) One hundred and thirty‐two triple‐negative breast cancer samples with mutation and RNA sequencing data are ordered by somatic mutation status. (B) Known cancer‐related genes^10^ that were mutated in at least 5.5% of the cases (upper) or differentially mutated per mRNA subtypes (lower). (C) Mutational signatures and (D) HRD score of the enrolled patients. Abbreviations: BLIS, basal‐like and immune‐suppressed; CEF‐T, fluorouracil, epirubicin, and cyclophosphamide followed by docetaxel; FUSCC, Fudan University Shanghai Cancer Center; HRD, homologous recombination deficiency; IM, immunomodulatory; LAR, luminal androgen receptor; MES, mesenchymal‐like; PCb, paclitaxel and carboplatin

**TABLE 1:** Clinicopathologic and genomic characteristics by treatment cohorts

	All patients (*n* = 132)		CEF‐T (*n* = 69)		PCb (*n* = 63)		
Characteristics	No.	%	No.	%	No.	%	*p* value
Age at diagnosis							
Median (IQR), years	53 (47–60)		52 (45–57)		54 (49–62)		0.83
Pathologic tumor size							
pT1	55	41.7	35	50.7	20	31.7	0.03
pT2–pT3	77	58.3	34	49.3	43	68.3	
Nodal status							
Negative	77	58.3	41	59.4	36	57.1	0.79
Positive	55	41.7	28	40.6	27	42.9	
Histological grade							
I–II	31	23.5	13	18.8	18	28.6	0.19
III	101	76.5	56	81.2	45	71.4	
Ki67 proliferation index							
≤14%	12	9.1	7	10.1	5	7.9	0.66
> 14%	120	90.9	62	89.9	58	92.1	
Surgery							
BCS	25	18.9	12	17.4	13	20.6	0.64
Mastectomy	107	81.1	57	82.6	50	79.4	
Adjuvant radiation							
Yes	58	43.9	28	40.6	30	47.6	0.42
No	74	56.1	41	59.4	33	52.4	
HRD score							
Median (IQR)	26.0 (–10.5 to 42.6)		16.3 (–16.2 to 38.1)		28.9 (2.8–48.0)		0.08
Intrinsic subtype							
Basal‐like	79	59.8	44	63.8	35	55.6	0.54
Others	24	18.2	11	15.9	13	20.6	
Unknown	29	22	14	20.3	15	23.8	
FUSCC subtype							
BLIS	35	26.5	20	29	15	23.8	0.70
IM	31	23.5	18	26.1	13	20.6	
LAR	23	17.4	10	14.5	13	20.6	
MES	14	10.6	7	10.1	7	11.1	
Unknown	29	22	14	20.3	15	23.8	
TP53							
Mutated	68	51.5	33	47.8	35	55.5	0.19
Wildtype	19	14.4	13	18.8	6	9.5	
Unknown	45	34.1	23	33.3	22	34.9	
PIK3CA							
Mutated	16	12.1	9	13	7	11.1	0.79
Wildtype	71	53.8	37	53.6	34	54	
Unknown	45	34.1	23	33.3	22	34.9	
KMT2C							
Mutated	8	6.1	2	2.9	6	9.5	0.14
Wildtype	79	59.8	44	63.8	35	55.6	
Unknown	45	34.1	23	33.3	22	34.9	
PTEN							
Mutated	6	4.5	2	2.9	4	6.3	0.42
Wildtype	81	61.4	44	63.8	37	58.7	
Unknown	45	34.1	23	33.3	22	34.9	

Subsequently, we investigated the predictive effect of HRD score and HRD‐related mutational signature. We found the interaction of HRD score and the two different chemotherapy regimens for RFS was significant (interaction *p* = 0.01), while there was no statistically significant interaction between HRD‐related mutational signature and different treatments (interaction *p* = 0.19). Then, patients were sorted by their HRD score value regardless of the regimen. In patients with values above the median, PCb was associated with significantly longer RFS compared with CEF‐T (Figure 2A hazard ratio [HR] = 0.30, 95% confidence interval [CI] 0.09–0.95, *p* = 0.03), while no evidence of different efficacy was found in the patients with lower HRD scores (Figure 2B HR = 1.53, 95% CI 0.59–3.96, *p* = 0.38). Consistently, within the PCb cohort, patients with HRD score above the median had a borderline significantly better RFS than the rest (HR = 0.36, 95% CI 0.11–1.19, *p* = 0.08), while no significant difference in RFS was detected in the CEF‐T cohort (HR = 1.81, 95% CI 0.72–4.53, *p* = 0.20).

**FIGURE 2:**
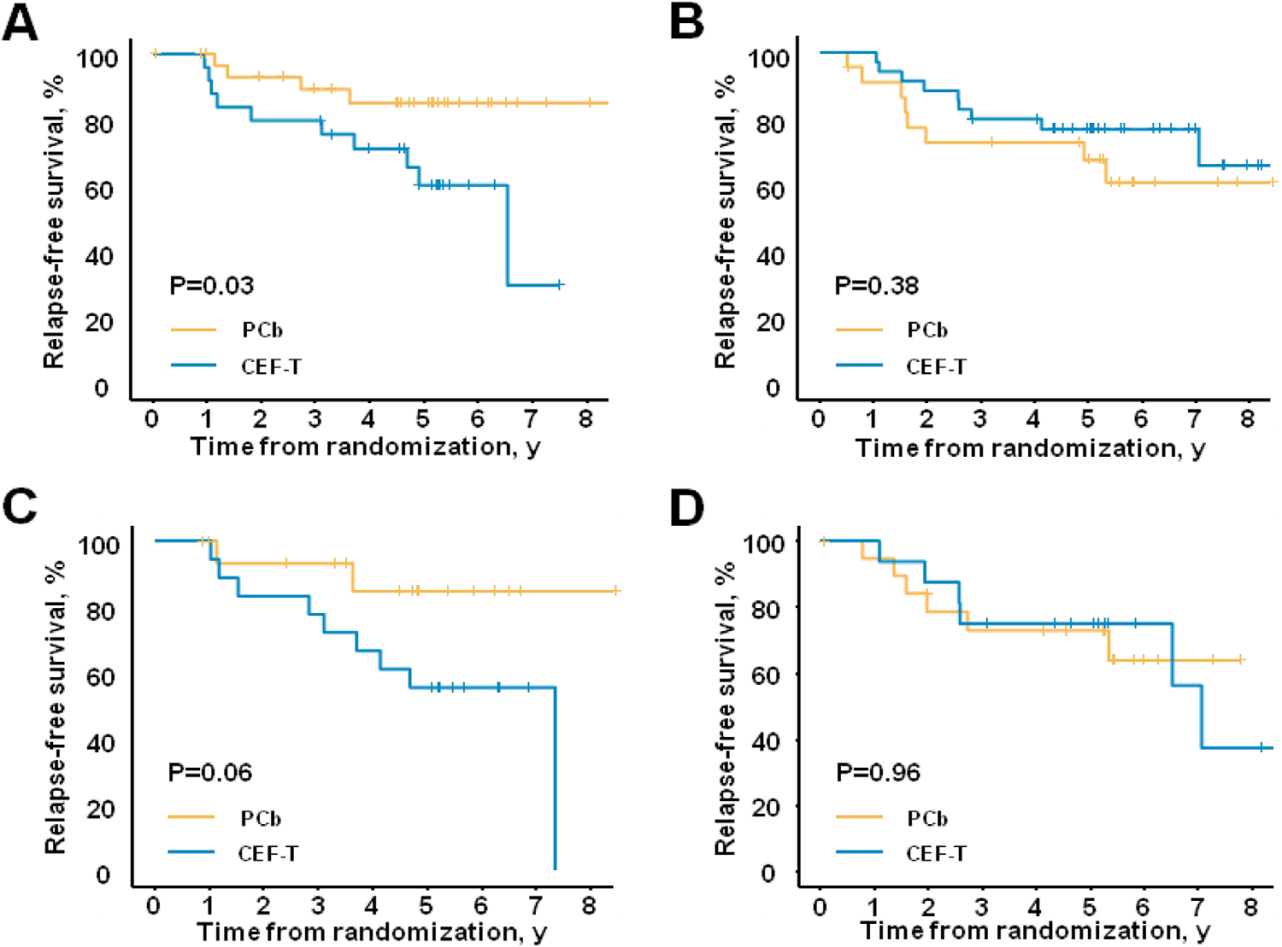
Survival analyses of potential biomarkers. (A) Survival analysis of patients with high HRD scores. (B) Survival analysis of patients with low HRD scores. (C) Survival analysis of patients with high HRD‐related mutational signature. (D) Survival analysis of patients with low HRD‐related mutational signature. Abbreviations: CEF‐T, fluorouracil, epirubicin, and cyclophosphamide followed by docetaxel; HRD, homologous recombination deficiency; PCb, paclitaxel and carboplatin

Although HRD is reversible, mutational signatures would not disappear, even if the defect is no longer active.^8^ Patients were also sorted by their HRD‐related mutational signature value regardless of the regimen. There was a borderline significant difference in RFS between the PCb arm and the CEF‐T arm in patients whose values were above the median (Figure 2C HR = 0.26, 95% CI 0.06–1.20, *p* = 0.06), while the outcome of the two cohorts was similar in the rest (Figure 2D HR = 0.97, 95% CI 0.31–3.04, *p* = 0.96). No significant difference in RFS was observed between the patients with values above the median and patients with values below the median in the PCb cohort (HR = 0.39, 95% CI 0.08–1.95, *p* = 0.24) or the CEF‐T cohort (HR = 1.61, 95% CI 0.56–4.63, *p* = 0.37).

Additionally, we also investigated the predictive effect of the intrinsic subtype and the FUSCC subtype. Multivariate analyses based on HRD score and HRD‐related mutational signature were also conducted, and the results were consistent with the univariate analysis (Table S3).

Furthermore, taking advantage of the RNA sequencing data, we found a couple of hypoxia and glycolysis‐related pathways associated with inferior prognosis in the CEF‐T cohort (Figure 3A). This finding was validated in the TNBC patients who received chemotherapy in METABRIC (Figure 3B). Moreover, upregulation of the gene set variation analysis score of Reactome glycolysis, a representative pathway regarding hypoxia and glycolysis, predicted inferior RFS in patients receiving CEF‐T (Figure 3C, left, HR = 3.43, 95% CI 1.37–8.60, *p* = 0.01), but not in those receiving PCb (Figure 3C, middle, HR = 0.47, 95% CI 0.10–2.23, *p* = 0.33). Association of Reactome glycolysis with worse RFS was also validated in the TNBC patients who received chemotherapy in METABRIC (Figure 3C, right, HR = 1.72, 95% CI 1.08–2.73, *p* = 0.02).

**FIGURE 3:**
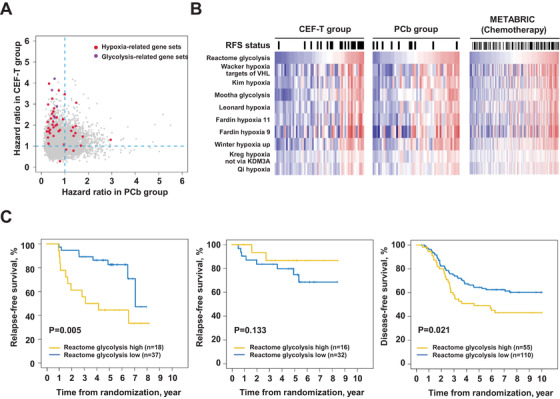
Expression of transcriptomic pathways and survival analyses. (A) Hazard ratio of the gene sets in the PCb and CEF‐T cohorts. (B) Patients in the PCb group and the CEF‐T group were ordered by a representative pathway concerning hypoxia and glycolysis. (C) Survival analysis of the Reactome glycolysis GSVA score in our CEF‐T cohort (left), PCb cohort (middle), and the METABRIC cohort (right). Abbreviations: CEF‐T, fluorouracil, epirubicin, and cyclophosphamide followed by docetaxel; GSVA, gene set variation analysis; METABRIC, Molecular Taxonomy of Breast Cancer International Consortium; PCb, paclitaxel and carboplatin

In our study, PCb was related to significantly longer RFS in patients with high HRD score, reflecting an adjuvant carboplatin‐containing regimen might bring more benefits to the TNBC population with high HRD status. By examining the HRD score, the candidate population for adjuvant carboplatin‐containing regimen can be expanded. Consistently, a borderline significant difference in RFS was detected between the two arms in patients with high HRD‐related mutational signature. Considering the limited number of cases involved, we believed that studies with a larger sample size are necessary to determine its effect.

Association between upregulation of glycolysis and hypoxia‐related pathways and inferior prognosis in the CEF‐T cohort was observed, and similar results were validated in METABRIC. Metabolic reprogramming is a major hallmark of tumor cells, and chemotherapy‐resistant TNBC cells usually display an enhanced glycolytic phenotype.^9^ Our findings suggest that tumor cells are possible to develop resistance to anthracycline/taxane regimen through transforming their expression of metabolic pathways. Thus, application of glycolytic inhibitors could become a potential treatment strategy to adopt.

In conclusion, the HRD score may serve as a biomarker to predict the efficacy of an adjuvant carboplatin‐containing regimen for TNBC. Upregulation of glycolysis and hypoxia‐related pathways was associated with inferior prognosis of patients treated by adjuvant anthracycline/taxane regimen. Whether metabolic alterations participate in resistance needs to be further studied, and relevant treatment strategies are worth exploring.

## FUNDING

Supported by grants from the National Natural Science Foundation of China (grants 81672600, 81722032, 82072916, and 91959207), the 2018 Shanghai Youth Excellent Academic Leader, the Fudan ZHUOSHI Project, the Municipal Project for Developing Emerging and Frontier Technology in Shanghai Hospitals (grant SHDC12010116), the Cooperation Project of Conquering Major Diseases in the Shanghai Municipality Health System (grant 2013ZYJB0302), the Innovation Team of the Ministry of Education (grant IRT1223), and the Shanghai Key Laboratory of Breast Cancer (grant 12DZ2260100).

## CONFLICT OF INTEREST

The authors declare that no conflict of interest exists.

## ETHICS APPROVAL AND CONSENT TO PARTICIPATE

The independent institutional review board of the participating centers approved the PATTERN study protocol. We performed the study according to the International Conference on Harmonisation Good Clinical Practice guidelines and ethical principles of the Declaration of Helsinki. All patients provided written informed consent.

## CONSENT FOR PUBLICATION

Consents for publication were obtained from all patients.

## DATA AVAILABILITY STATEMENT

Microarray data and sequence data were deposited in the NCBI Gene Expression Omnibus (OncoScan array; GSE118527) and Sequence Read Archive (WES and RNA‐seq; SRP157974). The data that support the findings of this study are available from the corresponding author upon reasonable request.

## Supporting information

Supporting information
