# Supplementary material for: Homologous recombination deficiency and glycolysis‐related pathway in adjuvant chemotherapy for triple‐negative breast cancer: A genomic landscape and biomarker assessment of the PATTERN trial
Source: Clin Transl Med. 2021 Sep 26;11(9):e513. doi: 10.1002/ctm2.513 (PMC8473643; doi:10.1002/ctm2.513)
Supplement: Supplementary file 1 — Supporting information [file CTM2-11-e513-s001.docx]

**Supplementary Content**

**Predictive effect of intrinsic subtype**

Breast cancer can be classified into different molecular intrinsic subtypes with different prognoses defined by mRNA expression of 50 genes (PAM50)^1^. Considering the association between BRCA1 mutation and basal-like breast cancer^2^, we subsequently explored the predictive effect of intrinsic subtypes. According to PAM50, 103 patients who underwent RNA sequencing assay were divided into basal-like type (79/103, 76.7%) and non-basal-like type (24/103, 23.3%). However, there was no significant difference in RFS between basal-like type and non-basal-like type no matter in the PCb arm (hazard ratio [HR]=1.88, 95% confidence interval [CI] 0.40-8.93, P=0.42) or in the CEF-T arm (HR=1.62, 95% CI 0.47-5.63, P=0.44). No significant difference in RFS was either detected in comparisons between the PCb cohort and the CEF-T cohort in patients of basal-like type (HR=0.71, 95% CI 0.30-1.66, P=0.42) or those of non-basal-like type (HR=0.58, 95% CI 0.10-3.45, P=0.54).

**Predictive effect of mRNA subtype**

Triple-negative breast cancer (TNBC) can be divided into four subtypes according to mRNA expression consisted of the following: the luminal androgen receptor (LAR) subtype, the immunomodulatory (IM) subtype, the basal-like and immune-suppressed (BLIS) subtype and the mesenchymal-like (MES) subtype^3^. Thus we also examine the predictive effect of the Fudan University Shanghai Cancer Center (FUSCC) subtype as a biomarker for treatment efficacy. However, no significant difference in relapse-free survival (RFS) was detected in comparisons between the paclitaxel-plus-carboplatin (PCb) cohort and the cohort using the regimen of cyclophosphamide/epirubicin/fluorouracil followed by docetaxel (CEF-T) in patients of all these four subtypes. (LAR: HR=0.81, 95%CI 0.16-4.02, P=0.80, IM: HR=0.80, 95%CI 0.15-4.45, P=0.80, BLIS: HR=0.52, 95%CI 0.16-1.68, P=0.27, MES: HR=0.74, 95%CI 0.07-8.22, P=0.80).

**Multivariate analysis and Subgroup analysis based on homologous recombination deficiency and mutational signature**

To confirm the reliability of the predictive effect of HRD score and HRD-related mutational signature, we conducted a multivariate analysis which also includes age of the patients, T stage, nodal status and histological grade. The results were shown in **Supplementary Table S3**.

Next, we divided the 67 patients who had both exome sequencing data and copy number detection data into four subgroups according to their status of homologous recombination deficiency (HRD) score and HRD-related mutational signature. Patients whose HRD score value was above the median were considered in high-HRD status and the rest were in low-HRD status. The mutational signature status was also divided following the same method. In this way, 21 patients with both high HRD score and mutational signature status were classified into high-high group and 19 in both low-HRD status and low mutational signature status were considered as low-low group. The high-low group has 16 patients with high HRD score and low mutational signature value. The remaining 11 patients were in low-high group whose features were relatively low HRD score and high mutational signature status. There was no significant difference in RFS between the PCb arm and the CEF-T arm in any of these four subgroups. No significant difference in RFS was detected in any comparison between these subgroups within the PCb cohort or the CEF-T cohort due to limited number of patients. Patients in low-low group did not show a better survival outcome compared to the other three subgroups in neither treatment arms.

Surprisingly, the high-high group has numerically worse prognosis compared to the other three subgroups in the CEF-T cohort (HR=2.44, 95%CI 0.77-7.69, P=0.12). Such relatively worse survival was also observed in the population with higher HRD score in the CEF-T cohort (HR=2.65, 95%CI 0.64-11.06, P=0.17). The similar trend was found in those who has greater value of mutational signature (HR=1.81, 95%CI 0.64-5.17, P=0.26). However, those differences are not significant, which may be partly caused by insufficient number of cases.

**Supplementary Table S1.** Characteristics of the PATTERN cohort and the patients undergoing multi-omic profiling

| Characteristics | Total | |  | CEF-T | |  | | PCb | |  |
| --- | --- | --- | --- | --- | --- | --- | --- | --- | --- | --- |
|  | Multi-omic  n=132 | PATTERN  n=647 | P value | Multi-omic  n=69 | PATTERN  n=322 | P value | | Multi-omic  n=63 | PATTERN  n=325 | P value |
| No. (%) |  |  |  |  |  |  |  |  |  |  |
| Age at diagnosis | 53 (47-60) | 51 (44-57) |  | 52 (45-57) | 50(44-57) |  | | 54 (49-62) | 51 (44-57) |  |
| Median(IQR),y |  |  |  |  |  |  | |  |  |  |
| Pathologic tumor size |  |  |  |  |  |  |  |  |  |  |
| pT1 | 55(41.7) | 351 (54.2) | 0.01 | 35(50.7) | 173(53.7) | 0.65 | | 20(31.7) | 178(54.8) | <0.01 |
| pT2-pT3 | 77(58.3) | 296 (45.8) |  | 34(49.3) | 149(46.3) |  |  | 43(68.3) | 147(45.2) |  |
| Nodal status |  |  |  |  |  |  |  |  |  |  |
| Negative | 77(58.3) | 481 (74.3) | <0.01 | 41(59.4) | 244(75.8) | 0.01 | | 36(57.1) | 237(72.9) | 0.01 |
| Positive | 55(41.7) | 166(25.7) |  | 28(40.6) | 78(24.2) |  |  | 27(42.9) | 88(27.1) |  |
| Histological grade |  |  |  |  |  |  |  |  |  |  |
| I-II | 31(23.5) | 177(27.4) | 0.36 | 13(18.8) | 88(27.3) | 0.14 | | 18(28.6) | 89(27.4) | 0.85 |
| III | 101(76.5) | 470(72.6) |  | 56(81.2) | 234(72.7) |  |  | 45(71.4) | 236(72.6) |  |
| Ki67 proliferation index |  |  |  |  |  |  |  |  |  |  |
| ≤14% | 12(9.1) | 80(12.4) | 0.29 | 7(10.1) | 40(12.4) | 0.60 | | 5(7.9) | 40(12.3) | 0.32 |
| >14% | 120(90.9) | 567(87.6) |  | 62(89.9) | 282(87.6) |  |  | 58(92.1) | 285(87.7) |  |
| Surgery |  |  |  |  |  |  |  |  |  |  |
| BCS | 25(18.9) | 203(31.4) | <0.01 | 12(17.4) | 99(30.7) | 0.03 | | 13(20.6) | 104(32.0) | 0.07 |
| Mastectomy | 107(81.1) | 444(68.6) |  | 57(82.6) | 223(69.3) |  |  | 50(79.4) | 221(68.0) |  |
| Adjuvant radiation |  |  |  |  |  |  |  |  |  |  |
| Yes | 58(43,9) | 296(45.7) | 0.70 | 28(40.6) | 144(44.7) | 0.53 | | 30(47.6) | 152(46.8) | 0.90 |
| No | 74(56.1) | 351(54.3) |  | 41(59.4) | 178(55.3) |  |  | 33(52.4) | 173(53.2) |  |

Abbreviations: BCS, breast conservative surgery; CEF-T, fluorouracil, epirubicin, and cyclophosphamide followed by docetaxel; IQR, interquartile range; PCb, paclitaxel and carboplatin.

| **Supplementary Table S2.** Characteristics of the PATTERN cohort and the METABRIC cohort | | | | | |
| --- | --- | --- | --- | --- | --- |
| Characteristics | PATTERN (n=132) | |  | METABRIC (n=165) | |
|  | No. | % |  | No. | % |
| Age at diagnosis |  |  |  |  |  |
| Median(IQR),y | 53 (47-60) | |  | 50 (41-59) | |
| Pathologic tumor size |  |  |  |  |  |
| pT1 | 55 | 41.7 |  | 55 | 33.3 |
| pT2-pT3 | 77 | 58.3 |  | 106 | 64.2 |
| Unknown | 0 | 0.0 |  | 4 | 2.4 |
| Nodal status |  |  |  |  |  |
| Negative | 77 | 58.3 |  | 26 | 15.8 |
| Positive | 55 | 41.7 |  | 131 | 79.4 |
| Unknown | 0 | 0.0 |  | 8 | 4.8 |
| Histological grade |  |  |  |  |  |
| I-II | 31 | 23.5 |  | 10 | 6.1 |
| III | 101 | 76.5 |  | 153 | 92.7 |
| Unknown | 0 | 0.0 |  | 2 | 1.2 |
| Surgery |  |  |  |  |  |
| BCS | 25 | 18.9 |  | 69 | 41.8 |
| Mastectomy | 107 | 81.1 |  | 90 | 54.5 |
| Unknown | 0 | 0.0 |  | 6 | 3.6 |
| Adjuvant radiation |  |  |  |  |  |
| Yes | 58 | 43.9 |  | 141 | 85.5 |
| No | 74 | 56.1 |  | 24 | 14.5 |

Abbreviations: BCS, breast conservative surgery; METABRIC, Molecular Taxonomy of Breast Cancer International Consortium; IQR, interquartile range.

**Supplementary Table S3.** Multivariate analysis of potential biomarkers

|  | PCb | | | CEF-T | | |
| --- | --- | --- | --- | --- | --- | --- |
| Variables | **HR** | **95% CI** | **P value** | **HR** | **95% CI** | **P value** |
| HRD score  (continuous) | 0.97 | 0.95-0.99 | 0.01 | 1.01 | 0.99-1.02 | 0.38 |
| Age  (continuous) | 0.91 | 0.84-0.99 | 0.02 | 1.01 | 0.96-1.06 | 0.70 |
| T stage  (pT1 versus pT2-3) | 2.06 | 0.49-8.70 | 0.33 | 1.30 | 0.45-3.78 | 0.63 |
| Nodal status  (negative versus positive) | 4.03 | 1.00-16.24 | 0.05 | 1.64 | 0.64-4.24 | 0.31 |
| Histological grade  (grade I-II versus grade III) | 5.58 | 0.95-32.73 | 0.06 | 0.79 | 0.20-3.06 | 0.73 |
| Variables | **HR** | **95% CI** | **P value** | **HR** | **95% CI** | **P value** |
| Mutational signature  (continuous) | 0.61 | 0.01-42.54 | 0.82 | 0.75 | 0.04-14.66 | 0.85 |
| Age  (continuous) | 0.95 | 0.84-1.07 | 0.41 | 1.04 | 0.98-1.09 | 0.20 |
| T stage  (pT1 versus pT2-3) | 0.84 | 0.11-6.15 | 0.86 | 0.31 | 0.07-1.39 | 0.13 |
| Nodal status  (negative versus positive) | 5.25 | 0.88-31.37 | 0.07 | 10.09 | 2.17-46.99 | <0.01 |
| Histological grade  (grade I-II versus grade III) | 0.67 | 0.11-4.25 | 0.67 | 0.28 | 0.07-1.21 | 0.09 |

Abbreviations: CEF-T, fluorouracil, epirubicin, and cyclophosphamide followed by docetaxel; CI, confidence interval; HR, hazard ratio; HRD, homologous recombination deficiency; PCb, paclitaxel and carboplatin.

**Supplementary Figure legends**

**Supplementary Figure S1** Schema of multi-omic profiling

**
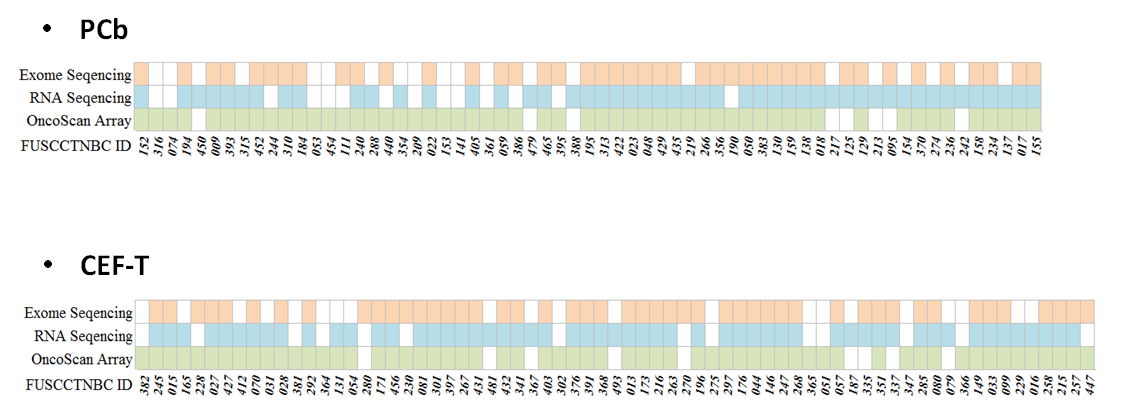
**

Colored squares indicate positive while blank squares indicate negative.

CEF-T indicates fluorouracil, epirubicin, and cyclophosphamide followed by docetaxel; FUSCCTNBC, Fudan University Shanghai Cancer Center Triple-Negative Breast Cancer; PCb, paclitaxel and carboplatin.

**References**

1. Parker JS, Mullins M, Cheang MC, et al. Supervised risk predictor of breast cancer based on intrinsic subtypes. *J Clin Oncol*. Mar 10 2009;27(8):1160-7.

2. Turner NC, Reis-Filho JS. Basal-like breast cancer and the BRCA1 phenotype. *Oncogene*. Sep 25 2006;25(43):5846-53.

3. Jiang YZ, Ma D, Suo C, et al. Genomic and Transcriptomic Landscape of Triple-Negative Breast Cancers: Subtypes and Treatment Strategies. *Cancer Cell*. Mar 18 2019;35(3):428-440 e5.
